# Effect of Ply Orientation on the Mechanical Performance of Carbon Fibre Honeycomb Cores

**DOI:** 10.3390/polym15112503

**Published:** 2023-05-29

**Authors:** Andrii Kondratiev, Václav Píštěk, Vitaliy Gajdachuk, Maksym Kharchenko, Tetyana Nabokina, Pavel Kučera, Ondřej Kučera

**Affiliations:** 1 Department of Materials Science and Engineering of Composite Structures, O.M. Beketov National University of Urban Economy in Kharkiv, Marshal Bazhanov Str. 17, 61002 Kharkiv, Ukraine; 2 Institute of Automotive Engineering, Brno University of Technology, Technická 2896/2, 616 69 Brno, Czech Republic; 3 Department of of Rocket Design and Engineering, National Aerospace University “Kharkiv Aviation Institute”, Chkalova Str. 17, 61070 Kharkiv, Ukraine; 4 3D METAL TECH LLC, Simi Khokhlovyh Str. 11/2, 04119 Kyiv, Ukraine

**Keywords:** thermo-dimensional stability, modulus of elasticity, finite element analysis, experimental prototype technology

## Abstract

Carbon fibres used as a honeycomb core material (subject to a proper in-depth analysis of their reinforcement patterns) allows solving the thermo-dimensional stability problem of the units for space systems. Based on the results of numerical simulations with the support of finite element analysis, the paper provides an evaluation of the accuracy of analytical dependencies for the determination of the moduli of elasticity of a carbon fibre honeycomb core in tension/compression and shear. It is shown that a carbon fibre honeycomb reinforcement pattern has a significant impact on the mechanical performance of the carbon fibre honeycomb core. For example, for honeycombs measuring 10 mm in height, the maximum shear modulus values corresponding to the reinforcement pattern of ±45° exceed the minimum values for a reinforcement pattern of 0° and 90° by more than 5 times in the XOZ plane and 4 times for the shear modulus in the YOZ plane. The maximum modulus of the elasticity of the honeycomb core in the transverse tension, corresponding to a reinforcement pattern of ±75°, exceeds the minimum modulus for the reinforcement pattern of ±15° more than 3 times. We observe a decrease in the values of the mechanical performance of the carbon fibre honeycomb core depending on its height. With a honeycomb reinforcement pattern of ±45°, the decrease in the shear modulus is 10% in the XOZ plane and 15% in the YOZ plane. The reduction in the modulus of elasticity in the transverse tension for the reinforcement pattern does not exceed 5%. It is shown that in order to ensure high-level moduli of elasticity with respect to tension/compression and shear at the same time, it is necessary to focus on a reinforcement pattern of ±64°. The paper covers the development of the experimental prototype technology that produces carbon fibre honeycomb cores and structures for aerospace applications. It is shown by experiments that the use of a larger number of thin layers of unidirectional carbon fibres provides more than a 2-time reduction in honeycomb density while maintaining high values of strength and stiffness. Our findings can permit a significant expansion of the area of application relative to this class of honeycomb cores in aerospace engineering.

## 1. Introduction

Modern structural materials and their design and technology concepts, as well as wide the possibilities of materials science and computer technologies, reveal unique reserves for improvements in the efficiency of structures in almost all areas of technology [1,2]. In particular, these reserves can be used in designing aerospace structures [3,4]. A specific feature of structures operating in open space, which are meant for the precision coordination of spacecraft interactions with ground objects, is the necessity to meet very stringent requirements, including the provision of their thermo-dimensional stability [5,6]. For example, the thermo-dimensional stability of the structural elements of spacecraft can be measured in fractions of a millimetre [7,8].

Sandwich structures with various types of cores (including honeycomb structures that are commonly used in flying vehicles) are known for their high thermo-dimensional stability [9]. The unique set of strengths, processes, and operational characteristics observed in honeycomb cores determined their extensive use and priority over other materials [10].

Honeycombs based on carbon fibre have been increasingly used in recent years, which allows significant improvements in the strength and stiffness of honeycomb structures and can extend their service life [11,12]. With the maximum specific strength and stiffness, a carbon fibre honeycomb core features the minimum coefficient of linear thermal expansion [13,14]. Combined with face sheets made of carbon fibre composites, the carbon fibre honeycomb core allows producing chemically homogeneous and dimensionally stable structures that are widely used in the aerospace industry [15,16]. A carbon fibre honeycomb core is a new structural material for aerospace engineering. In [17], a brief analysis of an application of 50 types of carbon-fibre-reinforced honeycomb composites was carried out. It is noted that one of the potential applications of this aggregate is its application in lightweight solar panels. A triaxial filler of the UCF-98-3/8-4.5 type was used on the X-33 apparatus. A carbon fibre honeycomb core was used as a baffle and lid in an LH2 tank. It is expected that this filler will also be used in antenna reflectors and other spacecraft components, which should provide high strength and thermal stability in operations.

Currently, there are many unsolved problematic tasks regarding improvements in the properties of carbon fibre honeycomb cores and structures [18,19]. For example, the use of carbon fibre composites as materials for honeycomb cores creates opportunities for regulating their consolidated physico-mechanical characteristics within a wide range by changing the reinforcing fibers’ laying angle to obtain an optimal combination of properties of the sandwich structure as a whole [20,21].

The optimal design of honeycomb composite structures requires the use of the consolidated physico-mechanical characteristics of cores [22,23]. Modern tools of engineering analyses allow for the direct finding of the stress–strain behaviour of such structures without a replacement of the core used by some solid orthotropic material, i.e., without its “smearing” [24,25]. However, the labour intensity of this approach, most likely, is justified only in cases where verification analysis is required [26,27]. In practical terms, designers have always used and continue to use analytical models of honeycomb structures, allowing them to theoretically calculate the consolidated physico-mechanical characteristics of cores, expressed via their geometric parameters and the properties of the material that they are made of [28]. The use of these models to obtain a deliberately approximate result mainly allows a further directive adjustment of the result [29]. From this, it follows that the use of analytical models and expressions implemented to determine physico-mechanical characteristics is a reliable and effective method for the optimization of the parameters of sandwich composite units for various applications, taking specific features of the structures into account [30,31].

Whereas these characteristics, expressed via the geometric and physico-mechanical properties of isotropic materials for the honeycomb cores and several other cores, were obtained long ago [28,29], similar consolidated characteristics of composite cores are not found in the literature. The bearing capacity of the panel and shell structures based on these materials is established in most cases by experiments [32]. For example, several companies produce honeycombs based on UCF-145-3/8-0.8- and UCF-121-1/4-3.0-grade carbon filler, and specific physico-mechanical characteristics are given below (Table 1) [33].

It is known that the values of the physico-mechanical characteristics of a honeycomb core significantly depend on the level of the operating load and the peculiar features of this load’s accommodation by individual elements of the honeycomb’s cell [34]. Attempts to achieve a satisfactory convergence of theoretical and experimental data related to the physico-mechanical characteristics of honeycomb cores are reduced to updating the known mathematical models of honeycombs [35] because of the impact of various technical deficiencies of a core [36].

The need for the continuous improvement of these structures contributes to active theoretical and experimental research aimed at the development of methods for designing this product class and the creation of experimental structures for operations in outer space [37].

The properties of the composite material and geometric parameters of the carbon fibre core were optimized in [38]. The paper shows that the compressive and shear strength of the core is well predicted by micromechanical fracture models. Technological methods for improving the shear performance of honeycomb cores reinforced with carbon fibre are described in [39]. Paper [40] proposes analytical models for the prediction of thermal conductivity and the strength of the composite core under compression, showing an acceptable agreement with the experiment. Paper [41] deals with the methods for reinforcing carbon fibre honeycombs, which provide higher strength due to the use of the curved wall topology technique and explores the effect of the geometric parameters of the core and characteristics of the composite materials used on the compressive strength. Analytical expressions for predicting the stiffness and strength of hexagonal carbon fibre honeycomb cores in transverse compression and shear are proposed in [42].

In most cases, depending on the criticality of honeycomb structures, a conclusion on the values of the given physico-mechanical characteristics of the core is given only based on the results of full-scale tests of honeycombs, which are rather expensive when a carbon filler is used for the honeycombs [43]. Paper [44] reviews the experimental results of the determination of the physico-mechanical characteristics of honeycombs made of various materials. It is shown that carbon fibre honeycombs feature a relatively stable linear stress–strain curve, and mechanical characteristics can be found based on the elastic model of the honeycomb’s behaviour. The experimental study of the effect of the compressive load on the behaviour of carbon fibre honeycomb cores with different cell shapes is conducted in [45]. Specimens of the cores were made by the corrugation of carbon fibre composite pieces with their subsequent gluing. Twenty-seven groups of specimens with different honeycomb cell shapes and thicknesses and varying heights with respect to the core were tested under the action of the transverse force. The results showed that the thickness of the cell was one of the main parameters affecting the mechanical performance of the cores, while the honeycomb’s height also has a minor impact on the mechanical performance of the cores. According to the results of the experiment, the density of the obtained carbon fibre honeycomb cores lies within the range of 157–282 kg/m^3^.

In connection with the above, analyzing the accuracy of analytical dependencies for the determination of the mechanical performance of carbon fibre honeycomb cores and their applicability at various stages of sandwich structure designs is an urgent task.

## 2. Materials and Methods

Analytical dependencies for the determination of the given physico-mechanical characteristics of a composite honeycomb core were obtained according to the pattern of a uniform distribution with respect to the volume of a typical honeycomb block element. These dependencies allow the theoretical calculation of the consolidated physico-mechanical characteristics of the cores, expressed via their geometric parameters and the properties of the composite material that they are made of. The characteristics of the composite were determined based on the mathematical models of the reinforcement theory. It is assumed that the reinforcing fibres of the composite material are laid symmetrically with respect to the middle surface of the package. The accuracy of analytical dependencies for the determination of the moduli of elasticity of the carbon fibre honeycomb core in tension/compression and shear, described by the adopted analytical dependencies, was assessed according to the results of a series of numerical simulations in the finite element analysis software ANSYS Mechanical 2021 R2. To determine the moduli of shear elasticity of the carbon fibre core with a regular hexagonal cell, shear tests conducted on twinned specimens using the stretching method were simulated using finite element analysis software. Modelling was carried out for 162 full cells of honeycombs. The finite element model was fixed along lateral unloaded plates for all linear displacements. Steel was taken as the material for the external plates of the shear test’s fixture models, with a central plate and edge plates measuring 10 mm and 5 mm thick, respectively. For the determination of the modulus of elasticity of a carbon fibre honeycomb core in transverse tension/compression, modelling was carried out on a single specimen using the finite element model described above. The external plate was loaded with a pressure of 0.1 MPa, with a fixation on the other plate for all linear displacements. A four-node multilayer quadrangular shell element exhibiting bending and membrane properties for spatial analyses (ShellL type and six degrees of freedom, including three translational and three rotational ones) was used in the process of generating a finite element grid. A study of the convergence of the numerical solution showed that normal and shear stresses varied slightly (by 5% at most) with this number of finite elements in the models. Analysis of the quality of the constructed finite-element models did not reveal any critical errors. The physico-mechanical characteristics of the carbon filler for honeycombs adopted for numerical simulations in the finite element analysis software corresponded to the KMU-4E material (manufacturer: Federal State Unitary Enterprise All-Russian Scientific Research Institute of Aviation Material, Moscow, Russia). Displacement patterns were used as boundary conditions in the process of finding the values of the given mechanical characteristics of the carbon fibre honeycomb core. Considering that the presence of edge effects causes the non-uniformity of these patterns, displacement values averaged over all nodes were used as the estimated values. The values of the moduli of elasticity were determined by standard methods for the considered test configurations.

During these studies, the technology and equipment for the manufacture of a carbon fibre honeycomb core with a cell face of 5 mm were developed. The technology is based on the block method. The used carbon fillers are listed below: unidirectional fibre of ELUR-P-A grade (manufacturer: Argon LLC, Balakovo-1, Moscow, Russia) impregnated with an ENFB epoxy binder (manufacturer: Federal State Unitary Enterprise All-Russian Scientific Research Institute of Aviation Material, Moscow, Russia) with a 0.13 mm thick monolayer; IMS-65 high-modulus carbon filler (manufacturer: Toho Tenax Co. Ltd., Tokyo, Japan) impregnated with an ENFB epoxy binder (manufacturer: Federal State Unitary Enterprise All-Russian Scientific Research Institute of Aviation Material, Moscow, Russia) with a 0.02 mm thick monolayer; sparse fibre of TC-36S-12K grade (manufacturer: TAIRYFIL, Congleton, UK) impregnated with an ENFB epoxy binder (manufacturer: Federal State Unitary Enterprise All-Russian Scientific Research Institute of Aviation Material, Moscow, Russia) with a 0.095 mm thick monolayer. The results of the experimental studies were obtained in the form of averaged values of the specific physical and mechanical parameters of six series of carbon-fibre-core pilot samples, and they were obtained in laboratory conditions using standard equipment, devices, and tools.

The validity of the conclusions is confirmed by using newly developed reliable mathematical models, and these models are compared with the results of numerical and experimental studies.

## 3. Theoretical Background

**Figure 1:**
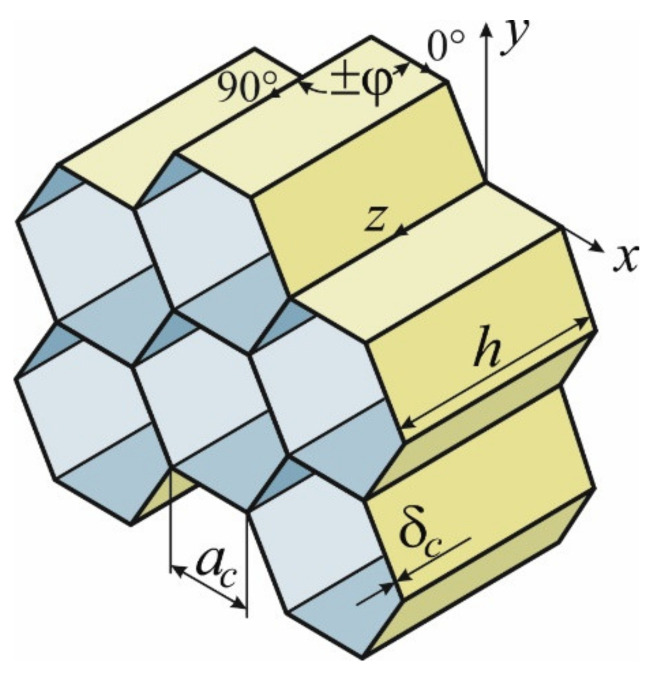
Adopted coordinate system and geometric parameters of the honeycomb core.

It is known [28,38] that the critical physico-mechanical characteristics of honeycomb cores are the moduli of elasticity in tension/compression in the $E_{z}$ direction transverse to the panel’s surface and shear moduli $G_{xz}\;\mathrm{and}$ $G_{yz}$ in the transverse direction (Figure 1).

The expressions for their theoretical definitions obtained in previous studies are widely used in the practice of designing honeycomb structures [28,29]. For a honeycomb core with a regular hexagonal cell, these dependencies take the following form:

(1) $$E_{z}=1.54\frac{\delta_{c}}{a_{c}}E_{m},\;G_{xz}=0.866\frac{\delta_{c}}{a_{c}}G_{m},\;G_{yz}=0.577\frac{\delta_{c}}{a_{c}}G_{m},$$

where $\delta_{c}$ and $a_{c}$ are the thickness and size of the honeycomb’s face, respectively; $E_{m}$ and $G_{m}$ are the modulus of elasticity and shear modulus of the honeycomb core’s filler material.

The use of the carbon filler as a material for honeycombs creates opportunities to vary their physico-mechanical characteristics within a wide range by changing the laying angle of reinforcing fibres into a symmetrical structure. The variation of the above angle in the interval of 0°≤φ≤90° allows obtaining an optimal combination of the stiffness properties of the honeycomb core. These dependencies should be completely valid for carbon fibre honeycombs as well if we substitute $E_{m}=E_{z\varphi }^{cm},$ $G_{m}=G_{xz\varphi }^{cm}$, and $G_{m}=G_{yz\varphi }^{cm}$ from the formulas in (1) into the adopted coordinate system (Figure 1).

The dependencies of the physico-mechanical characteristics of symmetrically reinforced composite materials representing a special case of dependencies that follow from the general mechanics of composites [46,47] are written as follows:

(2) $$E_{x\varphi }^{cm}=\frac{1}{\delta_{\Sigma }}\left(B_{11}-\frac{B_{12}^{2}}{B_{22}}\right);\;E_{z\varphi }^{cm}=\frac{1}{\delta_{\Sigma }}\left(B_{22}+\frac{B_{12}^{2}}{B_{11}}\right);\;G_{xz\varphi }^{cm}=\frac{B_{33}}{\delta_{\Sigma }};\;G_{yz\varphi }^{cm}=\frac{B_{33}}{\delta_{\Sigma }},\;$$

where the effective stiffness coefficients of the composite package should be as follows [48].

(3) $$B_{11}=\delta_{\Sigma }\left[{\bar{E}}_{1}\cos^{4}\varphi +2{\bar{E}}_{1}\mu_{21}\sin^{2}\varphi \cos^{2}\varphi +{\bar{E}}_{2}\sin^{4}\varphi +G_{12}\sin^{2}2\varphi \right];\;\;B_{12}=\delta_{\Sigma }\left[\left({\bar{E}}_{1}+{\bar{E}}_{2}\right)\sin^{2}\varphi \cos^{2}\varphi -G_{12}\sin^{2}2\varphi +{\bar{E}}_{1}\mu_{21}\left(\sin^{4}\varphi +\cos^{4}\varphi \right)\right];\;B_{22}=\delta_{\Sigma }\left[{\bar{E}}_{1}\sin^{4}\varphi +2{\bar{E}}_{1}\mu_{21}\sin^{2}\varphi \cos^{2}\varphi +{\bar{E}}_{2}\cos^{4}\varphi +G_{12}\sin^{2}2\varphi \right];\;B_{33}=\delta_{\Sigma }\left[\left({\bar{E}}_{1}+{\bar{E}}_{2}-2{\bar{E}}_{1}\mu_{21}\right)\sin^{2}\varphi \cos^{2}\varphi +G_{12}\cos^{2}\varphi \right];\;{\bar{E}}_{1}=\frac{E_{1}}{1-\mu_{12}\mu_{21}};{\bar{E}}_{2}=\frac{E_{2}}{1-\mu_{12}\mu_{21}}$$

Here, $E_{1},$ $E_{2},\;G_{12},\;\mathrm{and}\;\mu_{12}$ denote the elastic characteristics of the carbon-fiber-reinforced plastic (moduli of elasticity along and across fibres, shear modulus, and Poisson’s ratio, respectively); $\delta_{\Sigma }=\delta_{c}$ denotes the total thickness of the composite package. Poisson’s ratio $\mu_{21}$ can be determined from the experiment simultaneously with $E_{2}$ or from the condition of the existence of elastic potential $E_{1}\mu_{21}=E_{2}\mu_{12}$.

Let us consider the possibility of using dependencies (1) as related to the determination of the consolidated mechanical characteristics of the carbon fibre honeycomb core in order to reduce the costs and time for experimental or virtual research.

**Figure 2:**
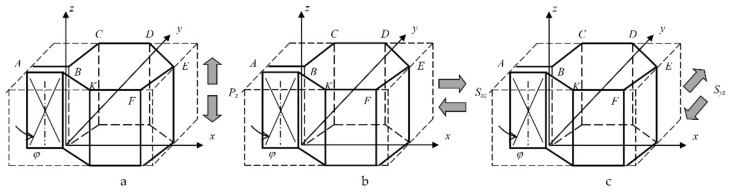
Representative element of the honeycomb core for the determination of its consolidated moduli of elasticity: (**a**)—$E_{z}$; (**b**)—$G_{xz}$; (**c**)—$G_{yz}$.

Figure 2 shows the representative element of the honeycomb core used for the determination of its consolidated mechanical characteristics according to the formulas in (1).

As shown in Figure 2, under the action of transversal forces $P_{z}$ on the representative element, all ends of the faces (*AB*, *BC*, *CD*, *DE*, *EF*, *FK* and *KB*) receive the same relative strain, $\varepsilon_{z},$ depending on reinforcing angle φ, which leads to the strict equality, $E_{z}$, of the honeycomb core relative to module $E_{z\varphi }^{cm}$ from Formula (2) within the plane of its face with a constant coefficient of $1.54\;\delta_{c}/a_{c}$.

Analysis of the element shown in Figure 2b allows establishing shear strain $\gamma_{xz}$ for the representative element of the honeycomb’s core, and it is strictly equal to these strains in the plane of the double *AB* face and faces *CD* and *FK.* The relative shear in four faces (*BC*, *DE*, *EF* and *KB*) should be equal to angle $\gamma_{xz}$ due to the observance of the law of parity for the tangential stresses in these faces and faces *AB*, *CD* and *FK*. Based on these considerations, the strict equality of $G_{xz}$ in the XOZ plane to shear modulus $G_{xz\varphi }^{cm}$ in the face planes with a constant coefficient of $0.866\;\delta_{c}/a_{c}$ should be valid.

Analysis of the honeycomb element in Figure 2c allows us to assume that shear strain $\gamma_{yz}$ for the representative element of the honeycomb core is strictly equal to those in fictitious faces *CK* and *DF* only. In this case, an assumption about the equality of honeycomb shear modulus $G_{yz}$ in the YOZ plane relative to shear modulus $G_{yz\varphi }^{cm}$ in Formula (2) with a coefficient of $0.577\;\delta_{c}/a_{c}$ requires experimental verification.

## 4. Numerical Implementation

Such verification was performed based on a series of numerical simulations with the use of finite element modelling. For the determination of the moduli of shear elasticity of the carbon fibre honeycomb core with a regular hexagonal cell, shear tests of twinned specimens by using the stretching method were simulated using finite element analysis software [49]. Modelling was carried out for 162 full cells of honeycombs.

**Figure 3:**
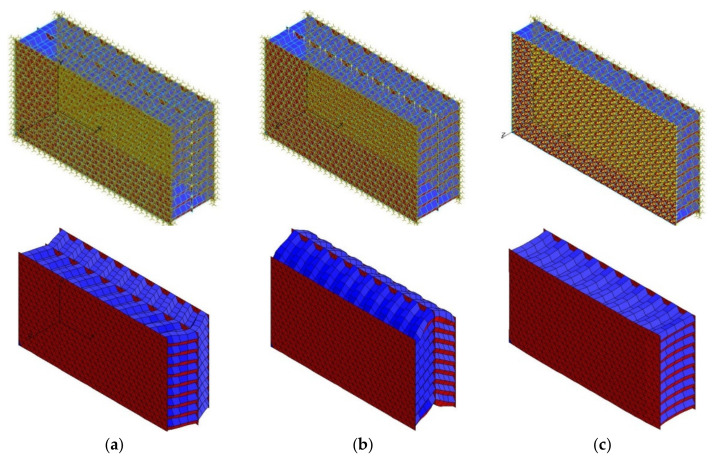
Generated finite element models of carbon fibre honeycomb specimens that are 10 mm high and corresponding deformed states: (**a**)—for determining the shear modulus $G_{xz}$; (**b**)—for determining the shear modulus $G_{yz}$; (**c**)—for determining the modulus of elasticity in transverse tension $E_{z}$.

In the process of determining shear modulus $G_{xz}\;$ in the *XOZ* plane, double faces of the specimen were oriented in the direction of the loading performed along the middle plate with a force of 200 N (Figure 3a).

To determine shear modulus $G_{yz}$ in the XOY plane, modelling was carried out on the same finite element model, but it was carried out using loading in a different direction along the middle plate with a force of 200 N (Figure 3b). The finite element model was fixed along the lateral unloaded plates for all linear displacements. Steel was used as the material for the external plates used in shear test fixture models, with a central plate and edge plates measuring 10 mm and 5 mm thick, respectively.

For the determination of the modulus of elasticity, $E_{z}$, of the carbon fibre honeycomb core in transverse tension/compression, modelling was carried out on a single specimen of the finite element model described above. The external plate was loaded with a pressure of 0.1 MPa, with a fixation on the other plate for all linear displacements (Figure 3c).

The physico-mechanical characteristics of the carbon filler for honeycombs adopted for further numerical simulations in the finite element analysis software are presented in Table 2 [33,50].

Figure 3 shows examples of the generated finite element models of specimens used to determine the moduli of elasticity of the carbon fibre honeycomb core, corresponding boundary conditions and deformed states of the virtual specimens obtained from the numerical simulations.

**Figure 4:**
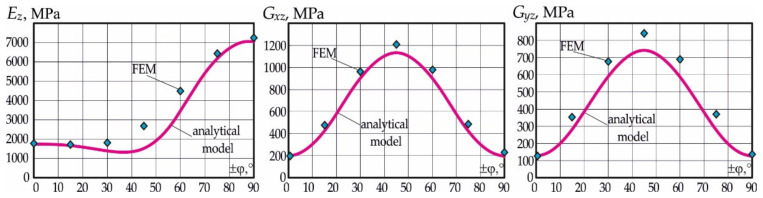
Graph of the dependency of the carbon fibre honeycomb core’s modulus of elasticity $E_{z}$ and shear moduli $G_{xz}$ and $G_{yz}$ on its reinforcing angle: 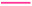 denotes analytical determination; 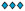 denotes data obtained from numerical simulations using finite element models.

Graphs in Figure 4 show the change in the moduli of elasticity of the carbon fibre honeycomb core, as calculated according to the formulas in (1), where the points obtained with the use of finite element analysis are plotted.

Analysis of the results allows us to establish the following. The values of both the modulus of elasticity and shear moduli $E_{z}^{CE}$, $G_{xz}^{CE}$ and $G_{yz}^{CE}$ obtained on the basis of the technologies of finite element analysis exceed the corresponding values determined by analytical Formula (1) $E_{z}^{A}$, $G_{xz}^{A}$ and $G_{yz}^{A}$. Such excess over the corresponding analytical values for shear moduli, close to the constant value for different reinforcing angles, is equal to $G_{xz}^{CE}/G_{xz}^{A}\approx 1.07$ and $G_{yz}^{CE}/G_{yz}^{A}\approx 1.14$. For the modulus of elasticity, $E_{z}$, the excess varies from 1.25 at φ=±30° to 1.8 at φ=±45°, and then it decreases again to 1.32 at φ=±60° and 1.03 at φ=±80°, which indicates some difference in the law of variation of $E_{z}$ from those found by analytical Formula (1).

## 5. Experimental Research

The technology and equipment for the manufacture of a carbon fibre honeycomb core with a cell face of 5 mm were developed in the process of research. The technology is based on the block method [33,50].

Primary blanks with a reinforcement pattern of ±45°, providing the maximum mechanical performance of a honeycomb core in shear, were formed using various carbon fillers. The used carbon fillers are listed below:

- Fibre of ELUR-P-A grade impregnated with an ENFB epoxy binder with a 0.13 mm thick monolayer;
- IMS-65 high-modulus carbon filler impregnated with an ENFB epoxy binder with a 0.02 mm thick monolayer;
- TC-36S-12K-grade sparse fibre impregnated with an ENFB epoxy binder with a 0.095 mm thick monolayer.

**Figure 5:**
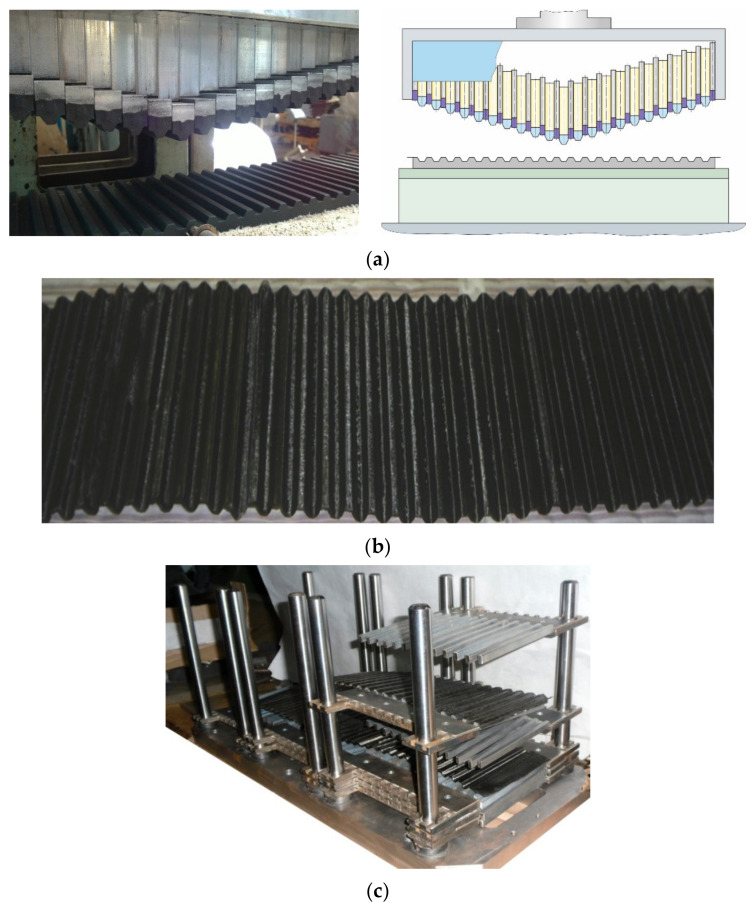
Equipment for the manufacture of a carbon fibre honeycomb core: (**a**)—forming punches; (**b**)—fragment of the corrugated blank; (**c**)—device for honeycomb gluing.

After a proper layup, the blanks were corrugated using a punch (Figure 5a). Corrugated blanks (Figure 5b) were assembled into a honeycomb block on the assembly fixture (Figure 5c).

**Figure 6:**
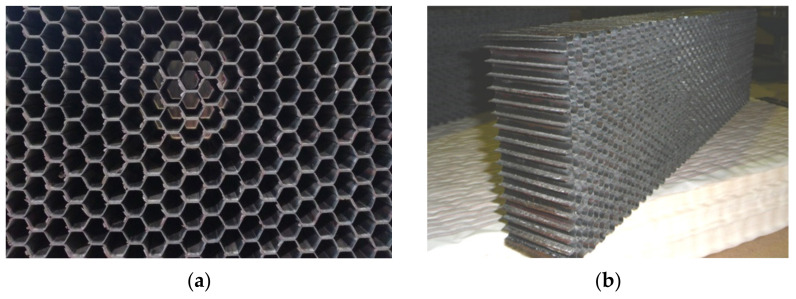
Fragment of the assembled honeycomb block with a cell size of 5.0 mm: (**a**)—before grinding; (**b**)—after grinding to a height of 60 mm.

After heat treatment, formed rods were removed from the assembled honeycomb block, cut around the perimeter (Figure 6a) and ground to the required height (Figure 6b).

Four series of specimens with a height of h=10 mm were made from the obtained blocks comprising carbon fibre honeycomb cores to determine their physico-mechanical characteristics.

Series 1 comprises a carbon fibre honeycomb core with a hexagonal honeycomb cell, where its wall contains two layers of unidirectional carbon fibres comprising 0.13 mm thick ELUR-P-A carbon tape impregnated with an ENFB epoxy binder. First, a specified even number of prepreg sheets was formed by laying and rolling on two layers of carbon tape with fibres oriented at an angle  of ±45° relative to the prepreg axis. The outer surfaces of both layers of the ELUR-P-A tape were duplicated with a fluoroplastic film that is 40 μm thick. Prepreg pressing was not performed. Prepreg was corrugated (Figure 5a) on a matrix preheated to 150 °C under a pressure of 0.3 MPa, and holding was carried out for 5–6 min. For this purpose, key punches with special metal tips were used. The corrugated prepregs were formed into a honeycomb block and cured using metal rods. At this time, BK-25 glue was applied onto the tangent surfaces of prepregs, and they were laid to provide the matching orientation of unidirectional carbon fibres on them. After that, the honeycomb block was cured in the oven with an increase in temperature at a rate of 0.8 to 1.2 °C/min: first to 130 °C, kept at that temperature for 30 min and then the temperature was raised to 175 °C at a rate of 1.8 to 2.2 °C/min; it was then held for 3 h at that temperature.

The resulting honeycomb core with walls formed from the unpressed prepreg process exhibited increased stiffness with respect to the double faces of the honeycomb cell due to the same orientation of unidirectional carbon fibres in their middle layers and the high-quality curing process, which is ensured by forming metal rods.

Series 2 comprises a carbon fibre honeycomb core with a hexagonal honeycomb cell, where its wall contains two layers of unidirectional carbon fibres comprising a 0.13 mm thick ELUR-P-A carbon tape impregnated with an ENFB epoxy binder. According to series 1, an even number of prepreg sheets were formed, and then they were pressed under a pressure of 0.5 MPa for 10 h at the shop’s temperature. The corrugation of prepregs, the application of BK-25 glue onto their tangent surfaces, the formation of a honeycomb block, and its curing were performed according to series 1.

The resulting honeycomb core also exhibited increased stiffness with respect to the honeycomb cells with walls that were formed by the pressed prepreg process, and this ensured the same orientation of unidirectional carbon fibres in the middle layers of the double cell faces and the high-quality curing process due to the formed metal rods.

Series 3 comprises a carbon fibre honeycomb core with a hexagonal honeycomb cell, where its wall contains four layers of unidirectional carbon fibres in the form of a unidirectional carbon tape made of a 0.02 mm thick IMS-65 tow that is impregnated with an ENFB epoxy binder. The prepreg was formed by rolling on carbon tape layers one by one, with the fibre orientation in the layers at an angle of ±45°;∓45° relative to the prepreg axis. Prepreg pressing was performed similarly to series 2, while prepreg corrugation, the application of BK-25 glue and the forming and curing of the honeycomb block corresponded to series 1.

Series 4 comprises a carbon fibre honeycomb core with a hexagonal honeycomb cell, where its wall contains four layers of unidirectional carbon fibres in the form of a nonwoven carbon tape made of a 0.02 mm thick IMS-65 tow that is impregnated with an ENFB epoxy binder. The formation, pressing and corrugation of prepregs and application of BK-25 glue on them were performed similarly to series 3. Corrugated prepregs were formed into a honeycomb block using silicone tubes. When the required number of rows of honeycomb cells is reached, the top matrix was placed on the upper corrugated prepreg, and the honeycomb block was clamped by an external clamping device. Then, the honeycomb block was placed into an oven, and excess pressure (0.7 MPa) was applied using air supply fittings to inflate the silicone shells to the size of honeycomb cells. Then, the honeycomb block was cured similarly to series 1. After cooling, pressure in the silicone shells was reduced to create a vacuum (0.08 MPa), and deflated silicone tubes were easily removed.

The resulting honeycomb core also featured increased stiffness with respect to the honeycomb cell wall and its double faces, which was ensured by the same orientation of unidirectional carbon fibers in their middle layers, and higher-quality curing with respect to all faces of the honeycomb cell due to use of formed silicone tubes.

## 6. Results and Discussion

Therefore, the above analysis proves that the application of analytical dependencies of the moduli of elasticity and shear moduli on the reinforcing angles as related to the carbon fibre honeycomb core is fully justified, in any case, at the early stages of the honeycomb structures’ design.

Table 3 presents the implementation results of a series of numerical simulations in finding the moduli of elasticity of the carbon fibre honeycomb core for the considered reinforcement patterns using fillers of various heights.

The findings indicate the following. The pattern of the reinforcement of a carbon fibre honeycomb core has a significant impact on its mechanical performance. For example, for a honeycomb with a height of h=20 mm, the maximum shear modulus values of honeycombs corresponding to a reinforcement pattern of ±45° exceed the minimum values for a reinforcement pattern of 0° and 90° by more than 5 times. The maximum modulus of elasticity of honeycombs in transverse tension, corresponding to a reinforcement pattern of ±75°, exceeds the minimum modulus for a reinforcement pattern of ±15° by more than 3 times.

We observed a decrease in the mechanical performance values of the carbon fibre honeycomb core depending on its height. With a honeycomb reinforcement pattern of ±45°, the decrease in the shear modulus is $\Delta G_{xz}\left(h\right)\approx 10$% in the XOZ plane and $\Delta G_{yz}\left(h\right)\approx 15\%$ in the YOZ plane at a honeycomb core height of h=10 mm. For the transverse tension, the reduction in the modulus of elasticity for a reinforcement pattern of ±75° is equal to $\Delta E_{z}\left(h\right)\approx 2\%$.

The results also indicate that the given maximum moduli of elasticity of the carbon fibre honeycomb core in tension and shear are provided by different reinforcement patterns with respect to the carbon filler. With reinforcement patterns of φ=±45° and φ=±75°, the honeycomb core has maximum reduced mechanical characteristics when in shear and tension, respectively.

**Figure 7:**
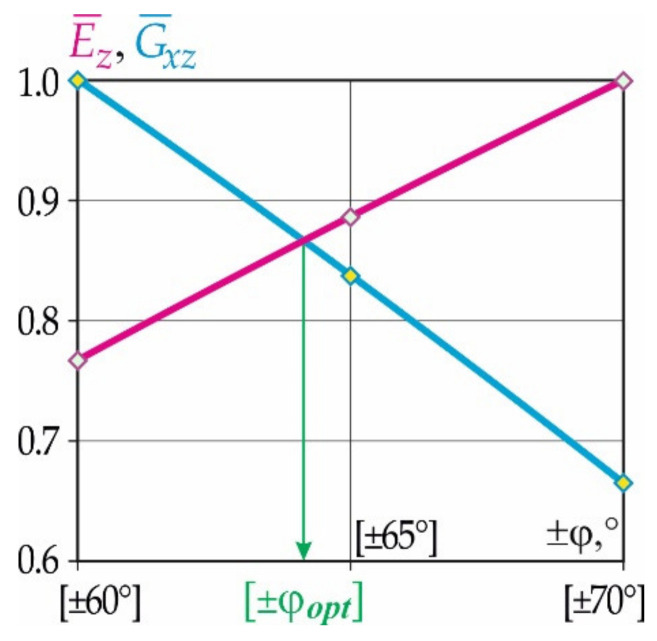
Graph of the dependency of the relative moduli of elasticity in transverse tension ${\bar{E}}_{z}$ and shear ${\bar{G}}_{xz}$ with respect to honeycomb reinforcement patterns close to rational values corresponding to the fixed height of a core of 10 mm: 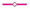 ${\bar{E}}_{z}$; 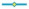 ${\bar{G}}_{xz}$.

The same high-level mechanical characteristics of the carbon fibre honeycomb core in transverse tension ${\bar{E}}_{z}=E_{z}/E_{zmax}$ and shear ${\bar{G}}_{xz}=G_{xz}/G_{xzmax}$ are possible only for the patterns of honeycomb reinforcements falling within a range of ±60°–±70°. A graph of the dependency of the relative moduli of elasticity in transverse tension ${\bar{E}}_{z}$ and shear ${\bar{G}}_{xz}$ with respect to the honeycomb reinforcement patterns close to rational values corresponding to the fixed height of the core is shown in Figure 7.

Analysis of the results indicates that a sufficiently high level of mechanical characteristics of the carbon fibre honeycomb core in both transverse tension and shear is provided by the reinforcement pattern of ±64°.

However, it is important to note that the accuracy of results of the numerical simulations is far from perfect because of their approximate nature. The reasons for the discrepancy, apparently, can be explained by the peculiarities of the consideration of the displacements in the nodes of the finite element model. For example, for the shear modulus at $G_{xz}$, φ=±45° and h=30 mm with averaging over all displacement nodes of the middle plate, $G_{xz}=1083$ MPa; in contrast, when calculated with the consideration of the maximum displacement, the same modulus is equal to $G_{xz}=865\;$ MPa, i.e., 0.8 of the previous value.

The results of experimental studies in the form of averaged values of the specific physical and mechanical parameters of four series of pilot samples of the carbon fibre honeycomb cores are given in Table 4.

Analysis of the data of series 1 and 2 showed that prepreg pressing allows an increase in the strength of the honeycomb core in shear to 10% in the XOZ and YOZ planes while the values of the moduli of elasticity in shear in the XOZ and YOZ planes also increased up to 13%.

The use of a larger number of thin-layer unidirectional carbon fibres (series 3) provided a 2.7 time reduction in the honeycomb volume weight while maintaining its strength and stiffness values.

With the same volume weight of the honeycomb core (series 4) but higher quality curing with respect to the corrugated prepregs, which was ensured by forming elastomer tubes, the shear strength of the honeycomb core in XOZ and YOZ planes increased by 7–9%, and the value of the moduli of elasticity in shear in the XOZ and YOZ planes increased by 7%.

The accuracy of maintaining the honeycomb cell’s geometric dimensions in all series was 0.05 mm; i.e., it was two times better compared to the prototype. The dimensional stability of honeycomb core blocks was assessed visually after conducting climatic tests by performing comparisons with a reference specimen, and a correspondence of their dimensional stability to the conditions of the operation of aerospace products was established.

As shown in Table 4, a significant disadvantage of the obtained carbon fibre honeycomb core is its large volume weight, which is predetermined by the thickness of the ELUR-P-A carbon tape.

Currently, the market for carbon fillers is developing rapidly, so new carbon fibres appear, which significantly surpass the carbon fibres produced earlier in terms of their physical, mechanical, thermo-physical and other characteristics [51]. Taking this fact into account, to form an experimental batch of the carbon fibre honeycomb core with a lower volume weight, two series of honeycomb specimens were produced. They were based on thinner prepregs and comprised high-modulus carbon filler TC-36S-12K and an ENFB epoxy binder with a 0.095 mm thick monolayer and reinforcement pattern of ±45° and ∓45°. Table 5 shows the averaged specific characteristics of honeycombs based on the filler used.

Analysis of the findings showed the following:

- The use of thinner fillers for the manufacture of carbon fibre honeycomb cores combined with the possibility of varying their reinforcement patterns allows controlling the physico-mechanical characteristics of this core within a wide range. Consequently, the use of a larger number of thin layers of unidirectional carbon fibres provided a more than 2-time decrease in the volume weight of the honeycomb while simultaneously maintaining its high strength and stiffness values (see the mechanical characteristics of the specimens of series 3, Table 4);
- The resulting mechanical characteristics are comparable to and in accordance with some strength indicators that are even higher than the relevant characteristics of similar UCF-121-1/4-3.0-grade honeycombs (Table 1);
- There is a potential to reduce the volume weight of carbon fillers to 12 kg/m^3^ (Table 4 and Table 5).

Therefore, the proposed and patented method [33,50] allows the manufacture of a carbon fibre honeycomb filler that exhibits precise and geometrically accurate hexagonal honeycomb cells, high shear stiffness and dimensional stability, meeting the conditions of the operation of aerospace products.

**Figure 8:**
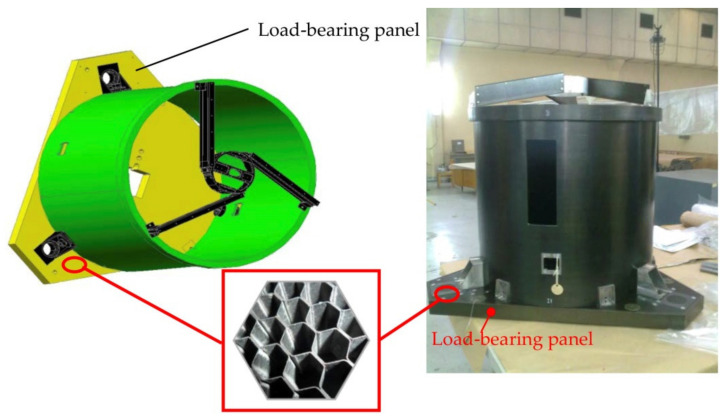
Application of the obtained carbon fibre honeycomb core in the load-bearing panel of the body of an optical, electronic module unit for an automatic spacecraft.

A productive step in the application of the obtained carbon fibre honeycomb core is its use in the body of an optical, electronic module unit for automatic spacecraft (Figure 8).

## 7. Conclusions and Further Research

Based on the results of numerical simulations with the support of finite element analyses, we evaluated the accuracy of analytical dependencies for the determination of the moduli of elasticity of a carbon fibre honeycomb core in tension/compression and shear, which are described by analytical dependencies (1)–(3). Their applicability at the initial stages of designing carbon fibre honeycomb structures for space applications is proven.

It is shown that the honeycomb reinforcement pattern has a significant impact on the mechanical performance of the carbon fibre honeycomb core. For example, for honeycombs with heights of 10 mm, the maximum shear modulus values corresponding to a reinforcement pattern of ±45° exceed the minimum values for a reinforcement pattern of 0° and 90° by more than 5 times in the XOZ plane and 4 times for the shear modulus in the YOZ plane. The maximum modulus of elasticity of the honeycomb core in transverse tension, corresponding to a reinforcement pattern of ±75°, exceeds the minimum modulus for a reinforcement pattern of ±15° by more than 3 times. We observed a decrease in the values of the mechanical characteristics of the carbon fibre honeycomb core, and this decrease was dependent on its height. With a honeycomb reinforcement pattern of ±45°, the decrease in the shear modulus is 10% in the XOZ plane and 15% in the YOZ plane. The reduction in the modulus of elasticity in transverse tension for the reinforcement pattern does not exceed 5%.

We determined a rational reinforcement pattern for the carbon fibre honeycomb core, providing a high-level modulus of elasticity and shear modulus. To simultaneously ensure high-level moduli of elasticity, it is necessary to focus on the reinforcement pattern of ±64°.

This paper proposes experimental prototype technology to produce carbon fibre honeycomb cores and structures for aerospace applications. Furthermore, the principles of new design and technology concepts related to obtaining efficient carbon fibre honeycomb core patterns that are aimed at reducing their density without compromising performance indicators have been developed, such as thermo-dimensional stability and physico-mechanical characteristics, and these are provided by the block method of cell formation.

A new method for the manufacture of carbon fibre honeycomb cores protected by our patent is described in this paper, and it differs in many ways from previously known methods and provides high core stiffness and strength and high dimensional and shape stability for cells, as confirmed by examples of the technology’s implementation and the six series tests of the pilot samples.

It is shown by experiments that the use of a larger number of thin-layer unidirectional carbon fibres provides a reduction of more than 2 times reduction in honeycomb density while maintaining high strength and stiffness values.

The technology for the manufacture of honeycomb structures based on carbon fibre honeycombs has been tested. Design and technology concepts have been developed for the load-bearing panel of the body of the optical, electronic module unit for automatic spacecraft.

Currently, the authors are engaged in further research on the optimization of the properties of carbon fibre honeycomb cores; the authors are also engaged in improving manufacturing technology. These endeavours will significantly expand the area of application of this class of honeycomb cores in aerospace engineering.

## Figures and Tables

**Table 1:** Specific physico-mechanical characteristics of carbon fibre honeycomb cores with a reinforcement pattern of ±30° produced by a number of companies.

Characteristics	UCF-145-3/8-0.8	UCF-121-1/4-3.0
Density, kg/m^3^	12.8	48.05
Specific compressive strength, km	2.07	5.73
Specific shear strength parallel to adhesive strips, km	1.35	4.41
Specific shear strength perpendicular to adhesive strips, km	0.77	2.71
Specific modulus of elasticity in shear parallel to adhesive strips, km	1042.	397
Specific modulus of elasticity in shear perpendicular to adhesive strips, km	659	279

**Table 2:** Physico-mechanical characteristics of the carbon filler for honeycombs adopted for further numerical simulations.

CFRP Grade	Prepreg Thickness (Monolayer), δ, mm	Modulus of Elasticity along the Fibres, $E_{1}$, GPa	Modulus of Elasticity across the Fibres, $E_{2}$, GPa	Shear Modulus $G_{12}$, GPa	Poisson’s Ratio, $\mu_{12}$	Density, ρ, kg/m^3^
CMU-4E	0.1	115	28.3	5.5	0.25	1550

**Table 3:** Results of the implementation of a series of numerical simulations to find the moduli of elasticity of the carbon fibre honeycomb core for the considered reinforcement patterns of fillers of various heights.

Size of Honeycomb Cell Face $a_{c}$, mm	Density, ρ, kg/m^3^	Honeycomb Reinforcement Pattern	Height of Honeycombs in Specimens h, mm	Modulus of Elasticity in Transverse Tension $E_{z}$, MPa	Modulus of Elasticity in Shear	
					$G_{xz}$, MPa	$G_{yz}$, MPa
5	97	0°; 90°	10	4520	235	201
			20	4511	221	165
			30	4505	211	149
		±15°	10	1712	478	354
			20	1688	447	310
			30	1671	427	289
		±30°	10	1808	962	678
			20	1699	900	611
			30	1624	858	565
		±45°	10	2679	1210	842
			20	2423	1137	770
			30	2269	1083	714
		±60°	10	4488	979	690
			20	4270	931	639
			30	4141	895	602
		±75°	10	6425	488	369
			20	6367	468	333
			30	6328	451	312

**Table 4:** Results of experimental studies in the form of averaged values of specific physical and mechanical parameters of four series of pilot samples of the carbon fibre honeycomb cores.

Series Number (Number of Layers × Layer Thickness in mm)	CFRP Material	Density, ρ, kg/m^3^	Specific Shear Strengths		Specific Moduli of Elasticity in Shear	
			$\tau_{xz}/g\rho ,$ km	$\tau_{yz}/g\rho ,$ km	$G_{xz}/g\rho ,$ km	$G_{yz}/g\rho ,$ km
1 (2 × 0.13)	ELUR-P-A + ENFB	117	5.69	3.52	562	317
2 (2 × 0.13)	ELUR-P-A + ENFB	117	6.25	3.8	630	358
3 (4 × 0.02)	IMS-65 + ENFB	40	6.3	3.9	650	422
4 (4 × 0.02)	IMS-65 + ENFB	40	6.9	4.18	660	452

**Table 5:** Specific mechanical characteristics of the produced experimental samples of a carbon fibre honeycomb core with a cell face of 5 mm based on thinner prepregs.

Material of the Honeycomb Core	TC-36S-12K + ENFB	Unit
Number of layers × layer thickness	2 × 0.095	mm
Honeycomb density ρ	85.0	kg/m^3^
Specific shear strengths: $\tau_{xz}/g\rho$	4.65	km
Specific shear strengths: $\tau_{yz}/g\rho$	2.93	km
Specific modulus of elasticity in shear: $G_{xz}/g\rho$	759	km
Specific modulus of elasticity in shear: $G_{yz}/g\rho$	517	km

## Data Availability

Not applicable.
